# A comprehensive spectral assay library to quantify the *Halobacterium salinarum* NRC-1 proteome by DIA/SWATH-MS

**DOI:** 10.1038/s41597-023-02590-5

**Published:** 2023-10-13

**Authors:** Ulrike Kusebauch, Alan P. R. Lorenzetti, David S. Campbell, Min Pan, David Shteynberg, Charu Kapil, Mukul K. Midha, Adrián López García de Lomana, Nitin S. Baliga, Robert L. Moritz

**Affiliations:** 1 Institute for Systems Biology, 401 Terry Ave N, Seattle, WA 98109 USA; 2 Center for Systems Biology, University of Iceland, Reykjavik, Iceland; 3 Departments of Biology and Microbiology, University of Washington, Seattle, WA USA; 4 Molecular and Cellular Biology Program, University of Washington, Seattle, WA USA; 5 Lawrence Berkeley National Lab, Berkeley, CA USA

**Keywords:** Proteomic analysis, Proteomics

## Abstract

Data-Independent Acquisition (DIA) is a mass spectrometry-based method to reliably identify and reproducibly quantify large fractions of a target proteome. The peptide-centric data analysis strategy employed in DIA requires *a priori* generated spectral assay libraries. Such assay libraries allow to extract quantitative data in a targeted approach and have been generated for human, mouse, zebrafish, *E. coli* and few other organisms. However, a spectral assay library for the extreme halophilic archaeon *Halobacterium salinarum* NRC-1, a model organism that contributed to several notable discoveries, is not publicly available yet. Here, we report a comprehensive spectral assay library to measure 2,563 of 2,646 annotated *H. salinarum* NRC-1 proteins. We demonstrate the utility of this library by measuring global protein abundances over time under standard growth conditions. The *H. salinarum* NRC-1 library includes 21,074 distinct peptides representing 97% of the predicted proteome and provides a new, valuable resource to confidently measure and quantify any protein of this archaeon. Data and spectral assay libraries are available via ProteomeXchange (PXD042770, PXD042774) and SWATHAtlas (SAL00312-SAL00319).

## Background & Summary

Proteins perform a diverse range of functions in every organism. Knowledge of protein abundance and the dynamic changes in a proteome are crucial to understand complex biological processes^1^. However, system-wide accurate and reproducible identification and quantification of peptides and proteins remains challenging. Mass spectrometry (MS)-based proteomics is continuously evolving as a sensitive and quantitative technique and can be broadly grouped in two acquisition strategies, discovery and targeted approaches. Data-Dependent Acquisition (DDA) is the preferred method to discover proteins in a sample, the method provides deep proteome coverage, especially in combination with pre-fractionation of samples, and identification is generally achieved by searching the data against a reference proteome. Though the stochastic nature of the DDA method and the dynamic range of proteins in complex matrices may lead to incomplete peptide measurements, particularly of low abundant peptide ions. Instead, targeted proteomics by Selected Reaction Monitoring (SRM) is highly reproducible across many samples but is limited to the detection of relatively few, predetermined sets of peptides that uniquely represent a set of proteins.

Data-Independent Acquisition (DIA) with its variant Sequential Window Acquisition of all Theoretical Mass Spectra (SWATH-MS) has become an established data acquisition strategy that provides benefits of both, traditional DDA and targeted SRM. DIA/SWATH-MS improves the detection and accurate quantification of large fractions of a proteome by combining deep proteome-coverage capabilities and high reproducibility across samples^2^, and its performance has been successfully benchmarked across laboratories^3^. In DIA/SWATH-MS, all peptide precursor ions within a pre-selected, relatively large mass isolation window are fragmented in an unbiased manner independent of their abundance. This generates a digital record of all ionized molecules, but also results in co-fragmentation of co-eluting ion species and highly complex MS/MS spectra that need to be deconvoluted by post-processing data analysis software. The analysis of DIA/SWATH-MS data relies typically on peptide-centric scoring strategies that require a spectral ion library, also referred to as DIA/SWATH or spectral assay library, to extract ion chromatograms in a targeted manner^2,4,5^. The quality and proteome coverage of an assay library are essential for obtaining accurate results^6^. Although libraries generated from DDA data provide best performance, recently untargeted library-free and hybrid strategies have been explored^7^. These approaches aim to detect peptides not included in a library while reducing the experimental effort, acquisition time and costs of generating libraries. Comprehensive, experimentally-derived spectral ion libraries have been generated for human^8^, mouse^9^, zebrafish^10^, *E. coli*^11^ and few other organisms.

*Halobacterium salinarum* NRC-1 (*H. salinarum*) is an extremely halophilic archaeon that requires high salt concentrations for cellular integrity and growth, and is widely found in hypersaline environments such as the Dead Sea and the Great Salt Lake, Utah, USA^12,13^. Originally isolated in salted fish a century ago^14^, *H. salinarum* became an extensively studied model organism^15,16^ that contributed to several notable discoveries such as bacteriorhodopsin^17^, the light-driven proton-pump that moves protons across the purple membrane out of the cell, or the first non-eukaryotic *N*- and *O*-glycosylated protein^18^ with its structural function^19^. *H. salinarum* added to our understanding of transcriptome architecture and genome reorganization^20^, DNA repair and photo-protection^21^, the role of gas vesicles^22^, and adaptions to extreme conditions and environmental challenges^23–26^. The team by Losensky *et al*.^27^ and Völkel *et al*.^28^ investigated biofilm formation of *H. salinarum* R1 by SWATH-MS with project-specific libraries based on the detection of 63% and 54% of the R1 strain proteome, respectively, and to the best of our knowledge these libraries are not publicly available.

**Fig. 1:**
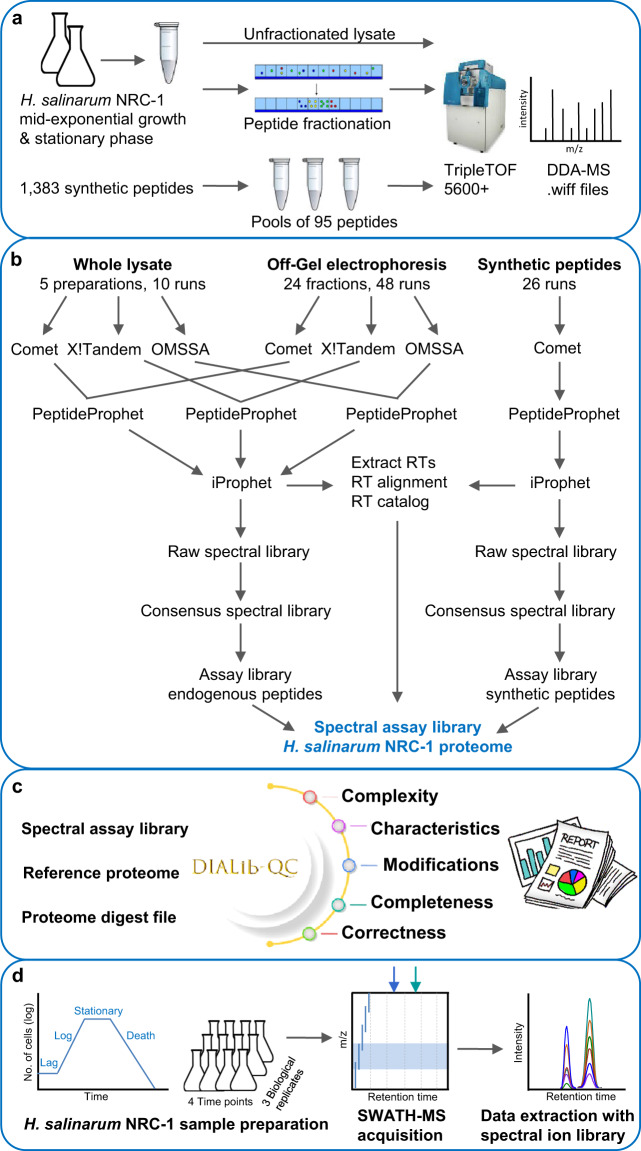
Schematic Overview. (**a**) Sample preparation for the *H. salinarum* NRC-1 library. *H. salinarum* NRC-1 grown to mid-exponential and stationary phase was subjected to data-dependent acquisition as unfractionated and fractionated digest. Endogenous samples were supplemented with synthetic peptide measurements for increased proteome coverage; (**b**) Library generation scheme. Data were searched with Comet, X!Tandem and OMSSA. Data were analyzed with the Trans-Proteomic Pipeline including PeptideProphet and iProphet followed by the generation of a raw, consensus, and spectral ion library; (**c**) DIALib-QC. The quality of the full spectral assay library and the 100 variable windows applied library was assessed with DIALib-QC, a software tool to evaluate libraries for defects and weaknesses; (**d**) Application of the *H. salinarum* NRC-1 spectral assay library. Analysis of *H. salinarum* NRC-1 collected at four time points of growth and in three biological replicates employing DIA/SWATH-MS and targeted data extraction using the developed *H. salinarum* NRC-1 100 variable window spectral assay library.

Here, we report a comprehensive, high quality spectral ion library of *H. salinarum* NRC-1 for the quantification of 21,074 distinct peptides that map to 2,563 *H. salinarum* NRC-1 proteins representing 97% of the predicted proteome. This library was generated from unfractionated and Off-Gel fractionated *H. salinarum* NRC-1 lysates grown to mid-exponential and stationary phase, and was supplemented with synthetic peptides to achieve nearly complete proteome coverage (Fig. 1a,b). The library has been evaluated with DIALib-QC^29^, a quality assessment tool for spectral ion libraries (Fig. 1c). We demonstrate the utility of this spectral assay library to identify and quantify the *H. salinarum* NRC-1 proteome collected at four time points of batch culture growth with three biological and technical replicates. We quantified 1,604 protein groups (1% FDR) (Fig. 1d) and compared the results to mRNA abundance determined from the same samples. The *H. salinarum* NRC-1 spectral assay library and associated data are publicly available at SWATHAtlas (www.swathatlas.org, SAL00312-SAL00319) and the PRIDE data repository (PXD042770^30^, PXD042774^31^), and we expect this library will advance quantitative proteome analyses in *H. salinarum* NRC-1 to further deepen our understanding of archaeal biology.

## Methods

### Cell culture

*H. salinarum sp*. NRC-1 (ATCC700922) was cultured in nutrient-rich complex medium: 250 g/L NaCl, 20 g/L MgSO_4_•7H_2_O, 3 g/L sodium citrate, 2 g/L KCl and 10 g/L peptone. Cultures were grown in unbaffled flasks with 40% of the flask volume occupied by the culture and illuminated at ~20 μmol/m^2^/s in Innova9400 incubators (New Brunswick). Cultures to generate the library were grown to mid-exponential growth (OD_600_ 0.55) and stationary phase (OD_600_ 1.27) at 37 °C under shaking at 220 rpm. Cells from 5 mL cultures were harvested by centrifugation at 8,000 × g for 2 min at 4 °C. The culture supernatant was discarded, pellets were snap-frozen on dry-ice/ethanol and stored at −80 °C.

Cultures for the time-course analysis were inoculated to a starting optical density OD_600_ 0.02 with starter culture of OD_600_ 0.5 derived from a single colony. Cultures were grown in triplicate and samples harvested at four time points: early exponential growth phase (OD_600_ 0.2; 14.3 h; TP1), mid- exponential growth phase (OD_600_ 0.5; 21.5 h; TP2), late exponential growth phase (OD_600_ 0.8; 28.8 h; TP3), and stationary phase (40.8 h; TP4). The final time point was selected to be 12 hours past the log-stationary transition since OD_600_ readings are not representative of cell growth in *H. salinarum* NRC-1 in stationary phase^32^. Cells at each time point were collected by centrifugation (8,000 × g, 2 min, 4 °C) for proteome analysis and RNA sequencing.

### Sample preparation

Cell pellets for both library generation and time-course analysis were resuspended in 750 μL Milli-Q water and disrupted at 4 °C using three 2.8 mm ceramic beads (Mo Bio Laboratories) and a Precellys 24 homogenizer (Bertin Corp) at 6500 rpm for 3 × 30 s followed by 6,800 rpm for 2 × 30 s, resting for 1 min between cycles. The protein content was determined by bicinchoninic acid assay (BCA) (Thermo-Fisher). Proteins were reduced with 5 mM Dithiothreitol (DDT, 45 min, 37 °C), alkylated with 14 mM iodoacetamide (IAM, 30 min, room temperature, darkness), followed by quenching of unreacted IAM with 5 mM DTT (15 min, room temperature, darkness). Samples were diluted 1:1 with 125 mM NH_4_HCO_3_ and digested with trypsin (Promega) at 1:50 enzyme:substrate ratio at 37 °C for 16 h. The digest was dried under centrifugal evaporation (Savant, Thermo-Fisher).

In addition, two mid-log phase samples (for library generation) were prepared with alternative methods: 1) 750 µL water with cOmplete EDTA-free protease inhibitor (Roche) were added to the cell pellet, the sample was vortexed and undissolved material removed by centrifugation at (16,000 × g, 10 min, 4 °C) followed by BCA, reduction, alkylation and digestion as described above, and 2) Cells were lysed with 750 µL water with cOmplete EDTA-free protease inhibitor using the Precellys 24 homogenizer as described above. The sample was centrifuged (16,000 × g, 10 min, 4 °C), the remaining pellet dissolved in 3 µL 10% SDS and combined with the soluble fraction. Proteins were reduced, alkylated and quenched as described above, and precipitated with 6 volumes of cold acetone (−35 °C, 16 h). The sample was centrifuged (3,000 × g, 10 min, 4 °C), the pellet resuspended in 125 mM NH_4_HCO_3_, the protein content determined by BCA and proteins digested with trypsin. Peptides were desalted with tC18 SepPak cartridges (Waters).

### Off-Gel electrophoresis (OGE)

Two cultures of *H. salinarum* NRC-1 grown to mid-log and stationary phase were subjected to pI-based peptide separation using the 3100 OFFGEL fractionator (Agilent Technologies). 400 µg of each sample was mixed and dissolved in OFFGEL stock solution (glycerol, ampholytes, water) according to the manufacturer’s protocol. Peptides were separated using immobilized pH gradient gel strips pH 3–10, 24 cm. Peptides were focused at 50 kVh with maximum current of 50 µA and a maximum voltage set to 8,000 V. Twenty-four in-solution fractions were collected, acidified with trifluoroacetic acid (pH < 2) and individually desalted using a tC18 96-well µ-elution plate (Waters). Samples were dried under centrifugal vacuum evaporation (Savant) and reconstituted in 0.1% formic acid/water. Each fraction was spiked with a set of synthetic peptides for retention time (RT) standardization^33^ prior to DDA analysis.

### Data dependent acquisition (DDA) for spectral assay library generation

Samples were analyzed with a TripleTOF® 5600 + equipped with a Nanospray-III® Source (Sciex) and an Ekspert™ nanoLC 425 with cHiPLC® system operated in trap-elute mode (Eksigent). Samples were loaded on a cHiPLC trap (200 µm × 500 µm ChromXP C18-CL, 3 µm, 120 Å) and washed for 10 minutes at 2 µL/min. Peptides were eluted on a nano cHiPLC column (75 µm × 15 cm ChromXP C18-CL, 3 µm, 120 Å) with 0.1% formic acid in water (A), 0.1% formic acid in acetonitrile (B) (v/v) using a gradient from 3% to 33% B in 120 min, 33%–63% B from 120–128 min and 63%–83% B from 128–132 min at a flow rate of 300 nL/min.

Data were acquired in DDA mode (also referred to as information dependent acquisition or IDA-MS/MS on Sciex MS instruments). A survey scan (TOF-MS) was acquired in the *m/z* range of 300–1,250 Da with 250 msec accumulation time. The 20 most intense precursor ions with charge state 2–4 above 300 counts per second were selected for fragmentation with rolling collision energy and a collision energy spread of +/− 15 V. MS/MS fragment spectra were collected in the range of 100–2,000 Da with 150 msec accumulation and a 3.3 s cycle time. Five lysate preparations and 24 OGE fractions were analyzed in technical replicates to generate a spectral assay library from endogenous samples (Table 1).

**Table 1:** Samples used to generate the *H. salinarum* NRC-1 spectral assay library.

Sample type	Peptide fractionation	MS samples	MS injections
Whole cell lysate	None	5	10
Whole cell lysate	OGE	24	48
Synthetic peptides	None	16	26
Total		45	84

Digested peptides from unfractionated and Off-Gel fractionated whole cell lysate were injected in technical replicates. Pools of synthetic peptides were injected once, and peptide subsets of some pools were injected again. OGE refers to Off-Gel electrophoresis.

### Synthetic peptides to increase the proteome coverage

For proteins not detected in the endogenous samples, two proteotypic peptide sequences per protein were selected, as far as possible, to develop assays with the use of synthetic peptides and the aim to increase the *H. salinarum* NRC-1 proteome coverage in the library. Likewise, for endogenous protein identifications based on a single peptide, an additional peptide sequence was selected. The *H. salinarum* NRC-1 PeptideAtlas^34^ was utilized to select peptides with empirical evidence or a predictive suitability score was calculated for candidate peptides. Then, the best proteotypic peptides for chemical synthesis and assay generation were determined following the criteria established in Kusebauch *et al*.^33^. For several peptides less stringent criteria (e.g., >25 amino acids, non-K/R C-terminus) were allowed as otherwise the respective proteins would have been excluded *a priori*. Being aware that these peptides will be challenging to detect or might fail, the aim was to make at least an attempt to represent these proteins in the library. A total of 1,383 peptides (Table ‘Synthetic peptide sequences’ deposited at PXD042770^30^) were individually chemically synthesized as free amine at the N-terminus and carboxylic acid at the C-terminus, cysteine residues were incorporated as carboxyamidomethylated cysteine building blocks (PEPotec SRM library Grade 1, Thermo-Fisher Scientific). Peptides were pooled in sets of 95, diluted to 5% acetonitrile, 0.1% formic acid in water (v/v), and 100 fmol of crude peptide were subjected to liquid chromatography tandem mass spectrometry (LC-MS/MS) and analyzed as described above.

### SWATH assay library generation

Instrument-native .wiff files acquired in DDA mode were converted to mzML using ProteoWizard msconvert^35,36^. MS/MS spectra were associated with peptide sequences using Comet (version 2014.01 rev. 0)^37^, X!Tandem (version Jackhammer)^38^ and OMSSA (version 2.1.8). The database comprised 2,646 *H. salinarum* NRC-1 protein entries (2,437 distinct protein sequences and 209 entries resulting from multiple genome mapping), a sequence-shuffled decoy counterpart, common contaminants and a set of peptides for retention time standardization. Searched peptides were allowed to be semi-tryptic with up to two missed cleavages. The search parameters included a fixed modification of +57.021464 for carbamidomethylated cysteine and a variable modification of +15.9949 for oxidized methionine. Variable N-terminal acetylation of +42.010565 was allowed in the Comet search. A monoisotopic mass error tolerance of 20 ppm was used in X!Tandem and Comet with the isotope error setting activated while 0.8 Da was used in OMSSA. The search results were processed and statistically validated with the open-source proteomics data analysis package Trans-Proteomic Pipeline^39,40^ (TPP, version 4.6.2) including PeptideProphet^41^ and iProphet^42^. Whole cell lysate and OGE data were processed together. Peptide spectrum matches (PSM) generated by each search engine were analyzed with PeptideProphet to assign each PSM a probability of being correct. The accurate mass binning and non-parametric model were enabled in the PeptideProphet analysis. Decoy hits were reported with a probability based on the model learned. PeptideProphet results were further processed with iProphet to refine the PSM-level probabilities and compute peptide-level probabilities based on corroborating information from the ensemble of identifications, and to combine the results from all three search engines. Next, a raw spectral library was built from iProphet results using SpectraST^43^ and a minimum probability threshold of 0.9 corresponding to a model-based error of 0.0023, followed by the generation of a consensus spectral library using up to 15 replicates per precursor ion. A SWATH assay library was generated from the consensus spectral library using the script spectrast2tsv.py available within the msproteomicstools software (https://pypi.python.org/pypi/msproteomicstools)^6,8^ with 300 to 1,250 *m/z* precursor mass range, 350 *m/z* as lower and 2,000 *m/z* as upper mass limits of fragment ions, theoretical masses, y- and b-ions, Peakview format and default settings for other parameters.

The same approach was taken to process the synthetic peptides, selected to supplement the *H. salinarum* NRC-1 library, using Comet as search engine. A spectral library from iProphet results with a minimum probability threshold of 0.9 corresponding to a model-based error of 0.0036 was generated for the synthetic peptide subset. The set of spiked-in synthetic peptides of human origin^33^, that were identified in each DDA run, were used by RTCatalog, a software tool part of the TPP^39,40^, to align retention times (RT) across all runs to build a retention time catalogue for the spectral assay library. First, all endogenous runs were aligned with the help of the spiked-in peptides using a linear-gradient correction, then all synthetic peptide measurements, and subsequently the synthetic peptides were aligned with the endogenous peptide RTs using their median RT values. Retention times of the same peptide sequences, sharing the same modifications, but of different charge states, were considered together when computing the statistics of the aligned RTs reported in the catalogue. Synthetic peptide assays (5% of the total library) were appended to the endogenous assay library to generate a comprehensive library of the *H. salinarum* NRC-1 proteome.

**Fig. 2:**
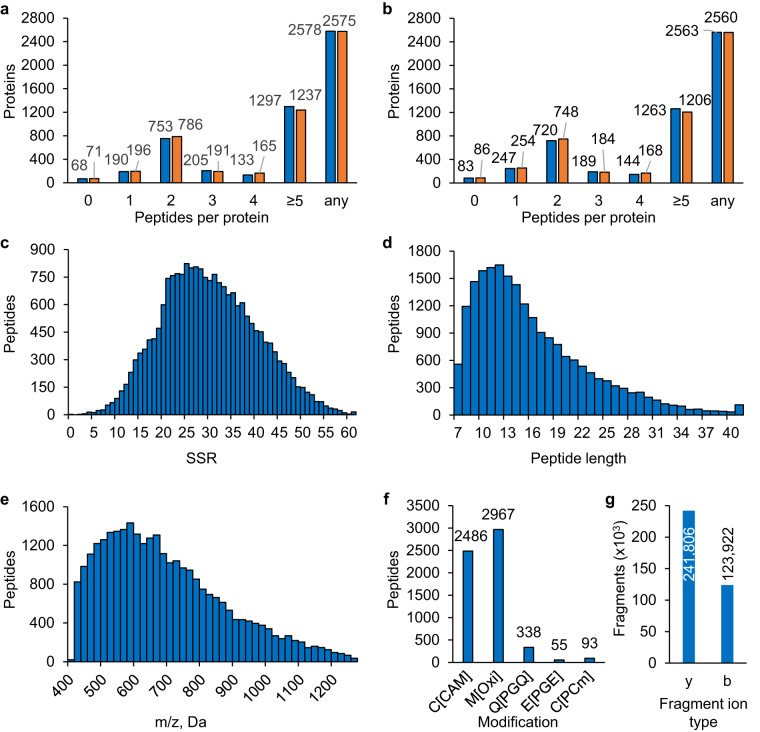
Coverage and characteristics of the *H. salinarum* NRC-1 spectral assay library. The graphs detail (**a**) the number of *H. salinarum* NRC-1 peptides per protein in the full SWATH assay library and (**b**) the number of peptides per protein in the 100 variable windows applied assay library. Blue depicts the coverage by any peptide mapping to the reference proteome including semi-tryptic peptides, and orange depicts the coverage by tryptic peptides only. ≥5 specifies five or more peptides, and “any” shows the number of proteins that are presented by at least one peptide. (**c**) to (**g**) depict values for the 100 variable windows applied library with (**c**) sequence specific retention (SSR) as a measure of relative hydrophobicity of peptides in the assay library, (**d**) distribution of peptide length, (**e**) distribution of precursor *m/z* across the acquired mass range, (**f**) type and occurrence of peptide modifications observed in the assay library with C[CAM] carbamidomethylation, M[Oxi] oxidation, Q[PGQ] pyroglutamate, E[PGE] pyroglutamatic acid and C[PCm] S-carbamoylmethylcysteine, and (**g**) frequency of observed b- and y- ion fragments with CID fragmentation.

Next, the same scheme of 100 variable acquisition windows (Table ‘100 variable SWATH windows’ deposited at PXD042770^30^, see also DIA/SWATH acquisition below) that was used to acquire the DIA/SWATH data was applied to this full DIA/SWATH ion library (HsalinarumNRC1_VNG_all_pv.txt) using the spectrast2tsv.py script. Further, transitions with a fragment ion mass within the isolation window of their precursor *m/*z were excluded to avoid interferences with incompletely fragmented precursors from the same SWATH window and y1, y2, b1 and b2 ions were removed due to their low specificity. For these reasons the 100 variable acquisition window library (HsalinarumNRC1_VNG_100vw_pv.txt) comprises a lower number of transitions compared to the unfiltered, full library (HsalinarumNRC1_VNG_all_pv.txt). Even though the full library includes more transitions, peptide numbers reported in the abstract and in Fig. 2c–g refer to the 100 variable acquisition window applied library which was used to analyze the DIA/SWATH-MS time-course data.

### Spectral assay library quality assessment

The quality of both spectral assay libraries was evaluated with DIALib-QC^29^, a software tool considering 62 parameters that highlight a library’s complexity, characteristics, modifications, completeness and correctness available online at http://www.swathatlas.org/DIALibQC.php (Fig. 1c). DIALib-QC assessment reports of the described assay libraries are provided in Table ‘DIALib-QC reports’ deposited at PXD042770^30^.

### DIA/SWATH acquisition of time-course samples

DIA/SWATH was performed on a TripleTOF® 5600 + equipped with a Nanospray-III® Source (Sciex) and an Ekspert™ nanoLC 425 with cHiPLC® system operated in trap-elute mode (Eksigent). Chromatography conditions were as described above for DDA acquisition. DIA/SWATH data were collected with a MS/MS^ALL^ SWATH™ acquisition method using 100 variable acquisition windows, each with a 1 Da overlap with the previous window (Table ‘100 variable SWATH windows’ deposited at PXD042770^30^). Q1 was scanned from 400–1,250 Da and MS/MS spectra were acquired from 100–1,700 Da with an accumulation time of 29 msec per SWATH window. A TOF-MS scan was acquired with 250 msec accumulation time for a total cycle time of 3.2 sec.

### DIA/SWATH data analysis with Spectronaut

DIA/SWATH data were processed with Spectronaut (version 14.10.201222.47784, Biognosys) and the developed SWATH 100 variable window assay library (HsalinarumNRC1_VNG_100vw_pv.txt) described above. Raw data files (.wiff) were converted to HTRMS format with the Spectronaut HTRMS converter (version 12.0.20491.2.35869). Default settings were used for the targeted data extraction. Briefly, data extraction mass tolerance (MS1 and MS2) was set to dynamic with a correction factor of 1, dynamic extracted ion chromatogram (XIC) RT window was enabled with a correction factor of 1 and local (non-linear) RT regression. Decoy assays were dynamically generated using the scrambled decoy method and library size fraction set to 1. Identification was performed using the normal distribution estimator with precursor and protein identification results with a q-value (false discovery rate (FDR)) cutoff of < 0.01. For quantification, interference correction was enabled, MS2 ion peak areas of quantified peptides were summed to estimate the protein peak areas, and area under the curve within integration boundaries as quantity type selected. Identified precursor quantities were normalized using the global normalization function (median) built-in Spectronaut. In condition setup, the four time points sampled in this study were defined as four conditions. For the directDIA analysis (library-free mode), the search database and parameter settings were kept the same as described above for the library-based analysis. Default settings were applied, with global normalization enabled. Trypsin specificity was set to allow for two missed cleavages, and precursor and protein identification results with a q-value (FDR) cutoff of <0.01 were employed.

### DIA/SWATH data analysis with OneOmics

DIA/SWATH data were processed within the OneOmics CloudOS environment (Sciex, https://multiomics.beta.sciex.com/sessions/sign_in). Study design (four time points, three biological replicates, three technical replicates) was defined in Experiment Manager. Extractor and Assembler were run with the following settings: 99% confidence filter, 999 peptides per protein, six transitions per peptide, RT calibration protein, 75 ppm XIC extraction width, ppm as XIC extraction unit. A confidence filter of ≥ 75% (statistically significant differentially expressed proteins) and most likely ratio (MLR) weight of ≥ 0.2 as measure of reproducibility^44^ were applied to report protein expression changes. P-values were determined within the OneOmics software environment by performing a t-test on the normalized weighted-average peptide areas for each protein across all samples in an experimental group. Abundance changes are reported relative to time point one.

### Principal component analysis

Sample grouping and reproducibility were determined using the OneOmics Principal Component Analysis (PCA) built-in function, which provided values for the two principal components with the highest observed experimental variance. We loaded the dataset into the R language environment and generated the visualization using the ggplot2 package^45^. In addition, a PCA analysis with the Spectronaut processed data was performed. Protein abundance values (q-value (FDR) < 0.01) were loaded into the R language environment. Protein group observations with missing values were excluded and the PCA was performed using the R stats::prcomp function with centering and scaling parameters enabled. The graphic representation was generated with ggplot2^45^.

### Proteome differential expression analysis

A proteome differential expression analysis for three time point contrasts, TP2 vs. TP1, TP3 vs. TP1, and TP4 vs. TP1, was performed using the OneOmics software. Three biological replicates (BR1, BR2, BR3) were taken into account to compute relative changes and significance parameters. Adjusted p-values were computed using the Benjamini-Hochberg method prior to filtering, and then filters to remove proteins not satisfying confidence ≥ 0.75 and MLR ≥ 0.2 were applied. Proteins satisfying criteria of |log2 (relative change)| ≥ 0.75 and adjusted p-value < 0.05 were considered “differentially expressed”. Graphical representations of relative change data, volcano plots and a heat map, were generated using ggplot2^45^ and ComplexHeatmap^46^ packages, respectively. Additionally, a heat map visualization with sample and protein group clusters for each time point using the Spectronaut protein group abundance data (q-value (FDR) < 0.01) was generated and the results visualized using ComplexHeatmap^46^.

### mRNA - protein abundance correlation analysis

Cells were cultured and collected as described above under *Cell Culture*. RNA sequencing (RNA-seq) raw data was obtained from de Lomana *et al*.^47^ (NCBI Bioproject PRJNA413990) and analyzed as described hereafter. Briefly, RNA was collected from cell lysate using Trizol-chloroform extraction and elution with water. Twelve barcoded libraries were prepared using the TruSeq Stranded mRNA HT library prep kit for mRNA. Libraries were pooled, denatured and diluted according to the NextSeq 500 protocol. Single-end sequencing of libraries was performed on the Illumina Nextseq500 platform using two high-output flow cells with 75 bp read lengths. Transcript abundance as transcripts per million was determined using kallisto^48^ and the *H. salinarum* NRC-1 non-redundant transcriptome sequence as reference to build the index. Protein and mRNA quantification data were quantile normalized and plotted using ggplot2^45^. To identify differentially expressed genes in this transcriptome dataset, we employed DESeq2^49^ with the estimated counts from kallisto (non-normalized data) as input. We considered genes with an |log2 (fold change)| ≥ 0.75 and an adjusted p-value of <0.05 to be differentially expressed.

## Data Records

### Data record 1

The raw mass spectrometry DDA files (.wiff and .wiff.scan), converted files (.mzML), results from the database searches (.pep.xml) and the search database (.fasta) used to generate the assay library have been deposited together with the consensus (.sptxt), DIA/SWATH assay libraries (.txt), and DIALib-QC reports (.pdf) to the ProteomeXchange Consortium^50^ (http://proteomecentral.proteomexchange.org) via the PRIDE partner repository^51^ with dataset identifier PXD042770^30^. Associated tables including ‘100 variable SWATH windows’, ‘Synthetic peptide sequences’ and ‘Unrepresented proteins in library’ are also deposited at PXD042770^30^. The spectral assay libraries and their DIALib-QC reports are also available at www.swathatlas.org (identifier SAL00312-SAL00319).

### Data record 2

The raw mass spectrometry DIA/SWATH files (.wiff and.wiff.scan) for quantifying the proteome at four different time points of growth including the data matrix obtained from the OneOmics analysis, the identified peptides and proteins as Spectronaut reports (.xls) using the developed spectral assay library as well as the directDIA approach have been deposited in ProteomeXchange and are accessible with dataset identifier PXD042774^31^.

## Technical Validation

### Generation of a comprehensive *H. salinarum* NRC-1 spectral assay library

Targeted DIA/SWATH and its peptide-centric data analysis strategy rely on high-quality spectral assay libraries. To accurately identify and reproducibly quantify peptides and their respective proteins we developed a spectral assay library from *H. salinarum* NRC-1 grown to mid-log and stationary phase. We measured trypsin digested lysates unfractionated and after pI-based peptide fractionation as well as 1,383 synthetic peptides (Fig. 1a). The acquired DDA-MS data (Methods and **Data Citation 1**) were searched against the *H. salinarum* NRC-1 reference proteome^34,52^ with three search engines for improved analysis and increased protein coverage^53^. Data were processed with the Trans-Proteomic Pipeline, a standardized suite of software tools for the analysis of MS/MS data, including PeptideProphet^41^ and iProphet^42^ for statistical validation of peptide spectrum matches (PSM) and distinct peptide identifications. A raw and a consensus spectral library were built using SpectraST^43^, then an ion library that includes the most intense and specific fragments as an assay list^6,8^ was developed from the *H. salinarum* NRC-1 consensus spectral library (Fig. 1b). The ion library derived from cell lysates comprised 1,924 proteins (73% of the *H. salinarum* NRC-1 reference proteome). For proteins that were not readily detected, e.g., proteins that are only expressed under certain conditions, we supplemented the ion library with assays developed from selected synthetic peptides (Table ‘Synthetic peptide sequences’ deposited at PXD042770^30^) aiming for increased proteome coverage. This resulted in a comprehensive spectral assay library with 408,795 transitions identifying 31,208 peptide precursor ions that represent 24,851 modified peptides, 21,983 stripped peptides and 2,578 *H. salinarum* NRC-1 proteins (excluding RT-Cal transitions, Table 2, Table ‘DIALib-QC reports’ deposited at PXD042770^30^, HsalinarumNRC1_VNG_all_pv.txt). While 97% of the peptides in the developed library are proteotypic, it includes 689 peptides that are shared among proteins, which is partly due to gene duplications and insertion sequences in the plasmids of *H. salinarum*^52^. With 1,288 of 1,383 selected synthetic peptides successfully developed into assays (93% success rate), synthetic peptides make 5% of the peptides in the *H. salinarum* NRC-1 spectral assay library.

**Table 2:** *H. salinarum* NRC-1 spectral assay library size.

	Proteotypic and Shared	Proteotypic
Proteins	2,578	2,258
Stripped peptides	21,983	21,355
Modified peptides	24,851	24,162
Precursors	31,208	30,350
Transitions	408,795	398,451

Overview of the number of *H. salinarum* NRC-1 proteins, stripped peptides, modified peptides, precursor ions and transitions that are included in the assay library (HsalinarumNRC1_VNG_all_pv.txt) considering all peptides, proteotypic and shared, and only proteotypic peptides. Reported numbers are excluding transitions used for retention time alignment.

Next, we applied 100 variable SWATH windows (Table ‘100 variable SWATH windows’ deposited at PXD042770^30^), a frequently used scheme of smaller Q1 windows in *m/z* dense regions and wider Q1 windows in sparse regions with fewer expected precursors, for improved specificity and acquisition coverage of complex samples. After removing fragment ions that fall into the SWATH window of the precursor and excluding small unspecific ions (y1, y2, b1, b2), the final 100 variable SWATH windows applied spectral assay library allows to target 365,536 transitions corresponding to 29,656 peptide ions, 23,842 peptides including modifications and 21,060 unmodified peptide sequences that represent 2,563 of 2,646 *H. salinarum* NRC-1 proteins (97% of the annotated proteome) (excluding RT-Cal transitions, Table ‘DIALib-QC reports’ deposited at PXD042770^30^, HsalinarumNRC1_VNG_100vw_pv.txt).

We then assessed the proteome coverage of the *H. salinarum* NRC-1 assay library in terms of peptides per protein for the full (Fig. 2a) and the 100 variable SWATH windows applied library (Fig. 2b). While 247 proteins in the 100 variable SWATH windows library (9% of the proteome) were observed by only one peptide, the majority of proteins (2,316 proteins, 88% of the proteome) in this extensive assay library is represented by two or more peptide assays and nearly half of the proteome (48%) by five or more peptides per protein (Fig. 2b, blue bars), demonstrating a deep proteome coverage of the *H. salinarum* NRC-1 proteome. The full library (Fig. 2a) provides a similar but slightly higher coverage than the 100 variable SWATH windows library (e.g., 49% of the proteome is represented by five or more peptides per protein) as it contains more transitions and peptides, and Q1 isolation windows and other filters are not applied as described above. Only 68 proteins of the *H. salinarum* NRC-1 proteome in the full library and 83 proteins in the 100 variable window assay library (3% of the proteome) remain unrepresented, mainly because these proteins have no suitable peptides that pass the selection and synthesis criteria (Table ‘Unrepresented proteins in library’ deposited at PXD042770^30^). Of note, 42 of the 68 unrepresented proteins in the full assay library are annotated as transmembrane proteins. Despite the known challenges of studying transmembrane proteins such as the tendency to aggregate or being insoluble in water, many of the transmembrane proteins not represented in the DIA/SWATH assay library are devoid of tryptic peptides between 7 and 50 amino acids in length with a relative hydrophobicity suitable for LC-MS analysis, and thus are not approachable by tryptic digestion even if solubility challenges are addressed.

Considering that 95% of the peptides in the *H. salinarum* NRC-1 assay library are derived from endogenous samples and may therefore include semi-tryptic peptides, we assessed the proteome coverage by fully tryptic peptides only. Although trypsin is a specific and efficient protease, semi-tryptic peptides occur as part of the proteolytic cleavage reaction. Yet, our results show equally good proteome coverage for the full and 100 variable SWATH windows library (Fig. 2a,b orange bars) with 2,560 proteins represented by fully tryptic peptides (97% of annotated proteome) and 2,306 proteins (87% of the proteome) by two or more peptide assays for the 100 variable SWATH windows library (Fig. 2b).

Next, we evaluated various assay library characteristics with DIALib-QC^29^ (Fig. 1c, Table ‘DIALib-QC reports’ deposited at PXD042770^30^) to ensure a high-quality library that supports accurate identification and quantification of peptides and proteins by DIA/SWATH data analyses. Figure 2c–g depict characteristics for the 100 variable SWATH windows library used for measuring global protein abundances over time (Fig. 1d). In the SWATH assay library 93.0% of the peptides have a sequence specific retention (SSR^54^ - a measure of relative hydrophobicity) of 10 to 46, a conservative hydrophobicity range that is readily detectable by most commonly used LC columns and gradients, and 99.9% of the peptides have an SSR value between 4 and 60, a more relaxed range including more hydrophilic and hydrophobic peptides that might be more challenging to detect depending on the LC conditions applied (Fig. 2c). The library contains peptides ranging in length from 7 to 51 amino acids, with 96% of the peptides between 7 and 30 amino acids, and an average peptide length of 16 amino acids (Fig. 2d), which is in great agreement with what has been observed for a developed *E. coli* assay library^11^. The 100 variable SWATH windows library comprises a precursor mass range of 400 to 1,250 *m/z* (Fig. 2e). Examining the type and occurrence of peptide modifications observed in the assay library, the largest group are 2,967 oxidized peptides (M[Oxi], + 15.9949 Da) as methionine is prone to oxidation during sample preparation, followed by 2,486 carbamidomethylated peptides (C[CAM], + 57.0214 Da), a modification of cysteine residues introduced by iodoacetamide during the alkylation step to avoid the formation of disulfide bonds. To a much smaller extent we observed the modification of N-terminal glutamine residues referred to as pyroglutamate (Q[PGQ], −17.0 Da) for 338 peptides, the cyclized modification of N-terminal glutamic acid residues (E[PGE], −17.0 Da) for 55 peptides, and S-carbamoylmethylcysteine cyclization at the N-terminus (C[PCm] (+39.994915) for 93 peptides (Fig. 2f). As expected with collision induced dissociation (CID) fragmentation, we observed a higher number of y fragment ions (66%) than b ions (34%) (Fig. 2g).

### Measuring global protein abundance changes by DIA/SWATH-MS

**Fig. 3:**
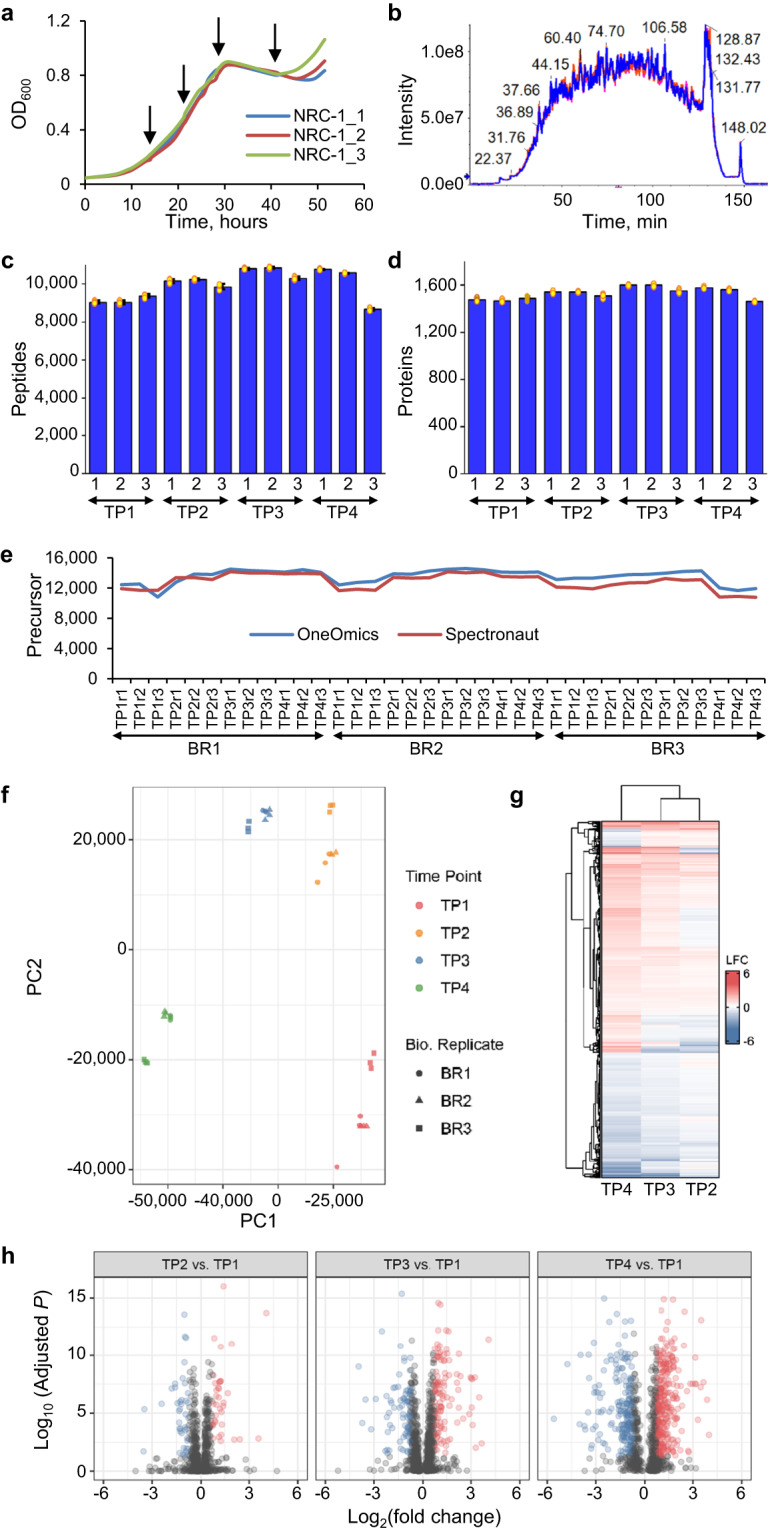
Measuring global protein abundance changes. (**a**) *H. salinarum* NRC-1 under standard growth conditions, three biological replicates are depicted in different colors and sampling time points are indicated at 14.3 h, 21.5 h, 28.8 h and 40.8 h; (**b**) Overlay of total ion current (TIC) of three SWATH-MS runs exemplifying technically reproducibility in data acquisition; (**c**) Number of unique peptides and (**d**) number of proteins for each biological replicate and time point identified with Spectronaut. The error bars indicate the variability within three replicates represented as standard error of the mean. These are calculated as the ratio of standard deviation of the number of quantified peptides or proteins observed in each biological replicate to the square-root of the sample size (n = 3). The yellow dots denote the number of identifications in each replicate and highlight consistency in identification at the peptide and protein level; (**e**) Overlap of identified precursor ions in each MS run determined with Spectronaut (red) and with OneOmics (blue) highlighting comparable identifications with both software tools; (**f**) Principal Component Analysis (PCA) of *H. salinarum* NRC-1 protein profiles. DIA/SWATH-MS analysis of total lysate shows that biological and technical replicates cluster tightly while data sampled at different time points separate from each other. PCA was performed within OneOmics. Each marker represents a SWATH-MS run. Marker shapes indicate biological replicates and colors highlight the sampled time points; (**g**) Heatmap of log2 fold protein abundance changes (LFC) obtained for three time point contrasts using OneOmics software. TP2 (mid-exponential phase), TP3 (late exponential phase), and TP4 (stationary phase) represent contrasts for each one of these time points in respect to early exponential phase (TP1). Data were visualized with ComplexHeatmap; (**h**) Volcano plots of differentially expressed proteins using OneOmics analyzed data and ggplot2 for visualization. Depicted are proteins satisfying criteria of |log2 (relative change)| ≥ 0.75 and adjusted p-value < 0.05.

To demonstrate the performance of the developed *H. salinarum* NRC-1 DIA/SWATH assay library, we measured global protein abundance changes over time under standard growth conditions and further compared these to the transcriptional time-course response. We sampled three biological replicates of *H. salinarum* NRC-1 at four defined time points including early exponential growth phase (14.3 h, TP1), mid-exponential growth phase (21.5 h, TP2), late exponential growth phase (28.8 h, TP3) and stationary phase (40.8 h, TP4) (Fig. 3a), and measured each sample in three technical replicates by DIA/SWATH-MS. The technical reproducibility in data acquisition is exemplified with biological replicate two (BR2) sampled at time point two (TP2) by overlaying the total ion current of the three technical replicates (Fig. 3b). RNA sequencing was carried out in parallel to the proteome analysis from the same samples.

DIA-MS analysis using the developed 100 variable window spectral assay library and Spectronaut software resulted in the identification of 8,629 to 10,895 distinct peptides (8,875 to 11,379 modified peptides) representing 1,396 to 1,527 protein groups (<1% protein FDR) in unfractionated cell lysate for each run (Fig. 3c,d). The number of detected peptides and proteins increased somewhat during exponential growth of *H. salinarum* NRC-1, followed by a slight decline in stationary phase. The median coefficient of variation (CV) of quantified precursors ranged from 9.7% to 11.5% in the sampled time points (Supplementary Fig. 1a). About 80% of all identified precursor ions showed a quantitative precision of ≤20% CV in each time point, except for TP4 with 75% of all precursor ions being quantified with ≤20% CV (Supplementary Fig. 1b). Across the four sampled time points of growth, we quantified 1,604 protein groups (<1% protein FDR, 1,682 *H. salinarum NRC-1* proteins, 1 RT-Cal protein) by the detection of 18,278 precursor ions from 13,846 distinct peptides corresponding to 63.6% of the annotated *H. salinarum* NRC-1 proteome or 65.6% of the proteome encompassed in the 100 variable windows spectral assay library. The quantified protein groups in each time point had a comparable dynamic range of four to five orders of magnitude (Supplementary Fig. 2).

The proteome coverage of 63.6% reached in this experiment slightly exceeds the proteome coverage in the *H. salinarum* NRC-1 PeptideAtlas^34^ reporting 62.7% of the predicted proteome from 88 combined experiments and thus demonstrates the achieved technology advancements. The *H. salinarum* NRC-1 PeptideAtlas also reports the detection of 188 out of 550 proteins with predicted transmembrane domains. Even though we were not able to develop assays for all transmembrane proteins (42 were not represented) we detected in this study remarkable 164 proteins with annotated transmembrane domains. Moreover, the 1,604 protein groups (1,682 *H. salinarum* NRC-1 proteins) quantified in this experiment are well in line with results of recent DIA/SWATH-MS analyses by Losensky *et al*.^27^ and Völkel *et al*.^28^ investigating biofilm formation in *H. salinarum* strain R1. Losensky *et al*.^27^ quantified 1,464 of 2,577 annotated *H. salinarum* R1 proteins (based on 176,510 transitions, estimated FDR of 1%) corresponding to 56.8% of the proteome^27^, and in a follow-up study looking into adaptions to heavy metal ion stress on the proteome level Völkel *et al*.^28^ reported the quantification of 1,180 proteins (<1% FDR) corresponding to 46% of the predicted *H. salinarum* R1 proteome^28^.

In addition to analyzing the data with Spectronaut, we also used the OneOmics software to demonstrate the developed ion library can be utilized with different DIA/SWATH software packages. With both software programs we identified comparable numbers of precursor ions, between 10,814 to 14,581 precursor ions per run with OneOmics and between 10,772 to 14,154 with Spectronaut, respectively (Fig. 3e), highlighting that the library works equally well with both tools.

To further evaluate the quality of our experimental data in context of sampled physiological states, we quantified replicate coherence using principal component analysis (PCA) within the OneOmics software. The first two principal components (PCs) show good reproducibility among time points and biological replicates, and PC1 performs well separating samples across the growth phase, which is depicted by biological replicates (BRs) clustering overall tight and technical replicates even tighter while different time points in growth differ from each other as expected (Fig. 3f). In comparison, BR1 and BR2 cluster very tight while BR3 measures slightly different to BR1 and BR2. Performing a PCA analysis using the data processed with Spectronaut, we confirmed the clustering for biological and technical replicates and separation of time points that we observed using the OneOmics processed data (Supplementary Fig. 3a).

Next, we assessed differentially expressed proteins of the *H. salinarum* NRC-1 proteome across physiological states. We computed relative protein abundance changes with respect to early exponential phase, i.e., log2 fold change of TP2 (mid-exponential phase), TP3 (late exponential phase), and TP4 (stationary phase) relative to TP1 (early exponential phase) with the OneOmics derived data. Using an unsupervised clustering-based approach, we observed physiological protein abundance changes during growth in batch culture and clustering by time point and biological replicates as depicted in Fig. 3g. In addition, we generated a heatmap with the Spectronaut processed data which likewise visualizes growth-phase associated protein abundance changes and a clustering by time point and biological replicates (Supplementary Fig. 3b). Further, we identified an increasing amount of differentially expressed proteins along the growth curve with 41 down regulated and 36 up regulated proteins in TP2 vs. TP1; 80 and 115 proteins in TP3 vs. TP1; and 198 and 308 proteins in TP4 vs. TP1, respectively (Fig. 3h).

While gene expression is a complex, multistep process and the relationship between mRNA and protein abundance is not trivial, across-gene analyses typically indicate a substantial correlation of mRNA and protein abundance, despite deviations^55,56^. In a previous study, we performed a systems-level interrogation of physiological changes of the *H. salinarum* NRC-1 transcriptome^32^. We reported, among other findings, four transcripts that showed transiently decreased abundance during exponential phase relative to abundance levels in early exponential and stationary phase, and transcripts of ten genes that are transiently elevated during exponential growth relative to early growth and stationary phase^32^. The developed *H. salinarum* NRC-1 assay library and acquired DIA/SWATH-MS data reported in this manuscript allowed to explore these observations on the proteome level. For several of these previously reported transient changes in transcript levels, we made the same observations by detecting protein abundance changes during exponential growth as exemplified for protein VNG6316G (ArcC), a carbamate kinase involved in arginine import and fermentation, which shows lower protein abundance during mid and late exponential growth relative to early exponential growth and stationary phase (Supplementary Fig. 4). An example for a gene transiently elevated during exponential growth is VNG0715G (ThiC), a protein associated with cofactor biosynthesis and thiamine diphosphate biosynthesis, for which we measured higher protein abundance in mid- and late exponential phase in comparison to early growth and stationary phase (Supplementary Fig. 5).

**Fig. 4:**
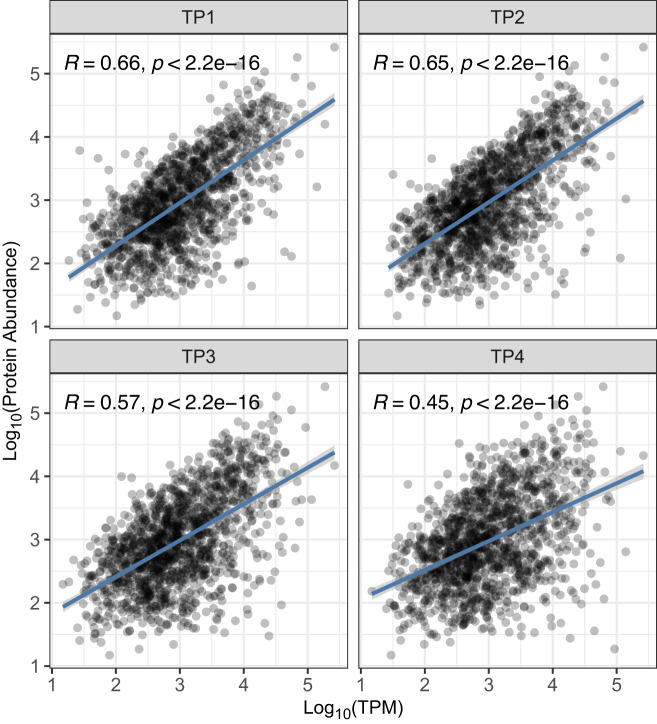
Correlation of protein and mRNA abundance. The plot depicts protein quantification (Spectronaut) in function of mRNA quantification (transcripts per million). Each panel represents a sampled time point with TP1 for early exponential phase (n = 1,315), TP2 for mid-exponential phase (n = 1,364), TP3 for late exponential phase (n = 1,404) and TP4 for stationary phase (n = 1,375). The dark blue solid line illustrates the fitted linear regression model and the shaded grey ribbon indicates its 95% confidence interval. R stands for the Pearson correlation coefficient and the p-value highlights the significant linear relationship between the quantitative variables.

Next, we assessed across-gene correlation of protein abundance measured with the 100 variable acquisition window library and mRNA abundance determined from the same samples. Our analysis shows reasonable correlation in TP1 (early exponential phase) and TP2 (mid-exponential phase) with R = 0.66 and R = 0.65, respectively (Fig. 4), while protein-mRNA abundance relationship scales somewhat less in TP3 (late exponential phase) and TP4 (stationary phase) with R = 0.57 and R = 0.45, respectively. Additionally, we observed that proteins that remained undetected during batch culture growth are overall associated with lower mRNA abundance in comparison to proteins that were detected and quantified in our study (p-value < 0.0001) (Supplementary Fig. 6). These results emphasize that we are able to verify the protein level observations reported with the developed assay library. The lower correlation in TP3 and TP4 is likely a result of post-transcriptional^57–59^ and post-translational regulation mechanisms^60^. We aimed to explore this in more detail in a recent study by integrating omics data from multiple sources and technologies to infer post-transcriptionally regulated genes and the putative mechanisms modulating their expression at the protein level in *H. salinarum* NRC-1^61^. Moreover, we successfully used the assay library to measure protein level changes in assembled ribosomal proteins investigating the interplay of transcriptional and translational regulation in *H. salinarum* NRC-1^47^. With the emerging availability of library-free DIA analysis methods, we also analyzed the time-course data with directDIA, a library-free analysis option within Spectronaut. For our data set, we observed more unique quantified peptides with the developed library in comparison to the library-free approach (19.7% vs. 14.4%). On the protein level, results are comparable, with the library-free mode reporting a slightly higher number of protein groups (10.0% vs. 7.8%), suggesting that a hybrid approach of the developed library in combination with directDIA may further increase the number of peptide and protein identifications (Supplementary Fig. 7). In conclusion, the DIA/SWATH spectral assay library presented here provides a valuable resource for the scientific community to rapidly identify and quantify nearly every *H. salinarum* NRC-1 protein in a variety of studies.

## Usage Notes

### Generating SWATH assay libraries with different Q1 isolation windows

To achieve a balance between sensitivity and specificity and ultimately improved data quality, the Q1 isolation window size can be altered based on the density of precursor masses in a sample^62^. In this study, we applied a scheme of 100 variable SWATH windows (Table ‘100 variable SWATH windows’ deposited at PXD042770^30^) to measure the *H. salinarum* NRC-1 proteome (HsalinarumNRC1_VNG_100vw_pv.txt). Depending on the goal of a study, any other window scheme can be applied to the full SWATH assay library (HsalinarumNRC1_VNG_all_pv.txt). We recommend that a library that has been modified with a different SWATH window isolation scheme should be assessed for conflict assays with DIALib-QC^29^. In any case, the SWATH window scheme for data acquisition needs to be identical with the SWATH window scheme applied to the library for data analysis purposes.

### Using different LC gradients and chromatography setups

To align retention times when using a different gradient length or chromatography set-up, the same synthetic peptides^33^ used for retention-time alignment in this study can be spiked in every sample or internal *H. salinarum* NRC-1 peptides can be used for retention-time alignment between runs. Another option is to use data analysis programs such as Spectronaut which support retention time alignment by iRT prediction when spike-in iRT peptides are not present in a sample.

### Spectral assay library portability

The generated *H. salinarum* NRC-1 assay library has been developed on a Sciex TripleTOF instrument and used for the analysis of data acquired on the same platform. While this is the preferred scenario, an assay library developed on a Sciex TripleTOF instrument can be used to analyze data collected on Thermo instruments as we previously demonstrated in Midha *et al*.^11^.

### Software compatibility for peptide centric DIA/SWATH analysis

The DIA/SWATH spectral assay libraries in this study are provided in PeakView format which is compatible with commonly used software programs for DIA/SWATH data analysis including PeakView, Spectronaut, OneOmics and Skyline with their recommended data analysis workflows. Other DIA analysis software may require a different import format of the library which can be converted by the user as usually the same parameters are required (e.g., precursor and fragment mass over charge, retention time, relative fragment intensity etc.) that are included in PeakView format.

## Supplementary information

Supplementary Information

## Data Availability

Code to perform PCA, generate heat maps and volcano plots, and carry out the transcriptome differential expression analysis is publicly available under repository https://github.com/alanlorenzetti/protDynContGenExp_v2.
