# Supplementary material for: A comprehensive spectral assay library to quantify the Halobacterium salinarum NRC-1 proteome by DIA/SWATH-MS
Source: Sci Data. 2023 Oct 13;10:697. doi: 10.1038/s41597-023-02590-5 (PMC10575869; doi:10.1038/s41597-023-02590-5)
Supplement: Supplementary file 1 — Supplementary Information [file 41597_2023_2590_MOESM1_ESM.pdf]

SUPPLEMENTARY INFORMATION

A comprehensive spectral assay library to quantify the *Halobacterium salinarum* NRC-1 proteome by DIA/SWATH-MS

Ulrike Kusebauch[1], Alan P. R. Lorenzetti[1], David S. Campbell[1], Min Pan[1], David Shteynberg[1], Charu Kapil[1], Mukul K. Midha[1], Adrián López García de Lomana[1], Nitin S. Baliga[1,2,3,4] Robert L. Moritz[1]*

[1] Institute for Systems Biology, Seattle, WA, USA

[2] Departments of Biology and Microbiology, University of Washington, Seattle, WA, USA

[3] Molecular and Cellular Biology Program, University of Washington, Seattle, WA, USA

[4] Lawrence Berkeley National Lab, Berkeley, CA, USA

*Corresponding author: Robert L. Moritz (rmoritz@systemsbiology.org)

Table of contents

Supplementary Figure 1. Coefficient of Variation (CV)……………………………..………………3

Supplementary Figure 2. Dynamic range……………………….…………….....…………………4

Supplementary Figure 3. Proteome profiles of *H. salinarum* NRC-1 measured with DIA/SWATH-MS…………………………………………………………………………………...…………..5

Supplementary Figure 4. VNG6316G shows transiently decreased protein abundance during exponential growth……………………………………………………………………………………..6

Supplementary Figure 5. VNG2604Gm shows transiently elevated protein abundance during exponential growth……………………………………………………………………………………..8

Supplementary Figure 6. mRNA abundance levels of detected and undetected proteins……10

Supplementary Figure 7. Library-based and library-free analysis...……………………………..…11

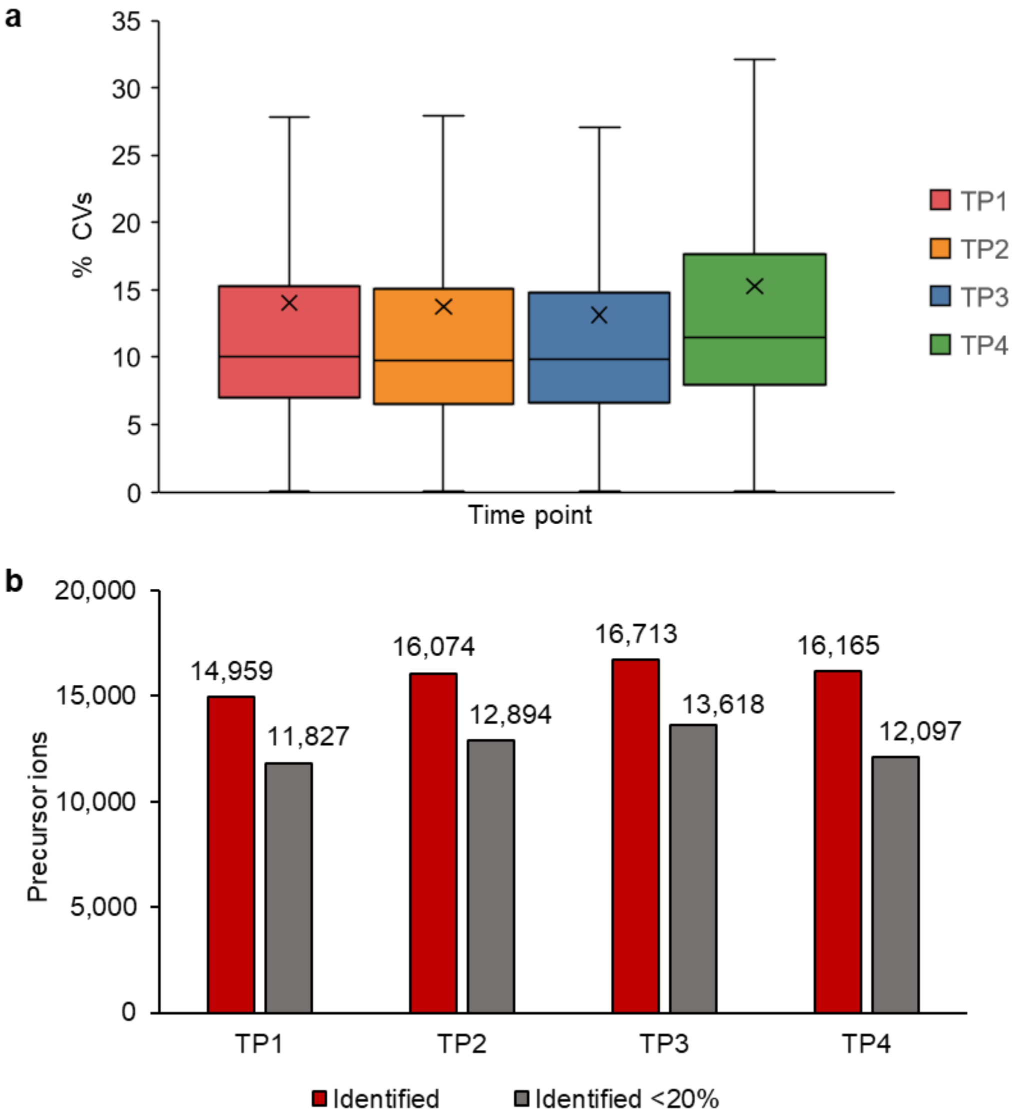

Supplementary Figure 1. Coefficient of Variation (CV). **(a)** The plot depicts the % CV distribution of quantified precursors for each sample time point (TP) with the solid line indicating a median % CV of 10.1% for TP1, 9.7% for TP2, 9.8% for TP3 and 11.5% for TP4. The x symbol in the plot denotes the mean % CV; **(b)** The graph displays the number of all precursor identifications regardless of quantitative precision (red) and the number of precursors below 20% CV (grey) for each time point.

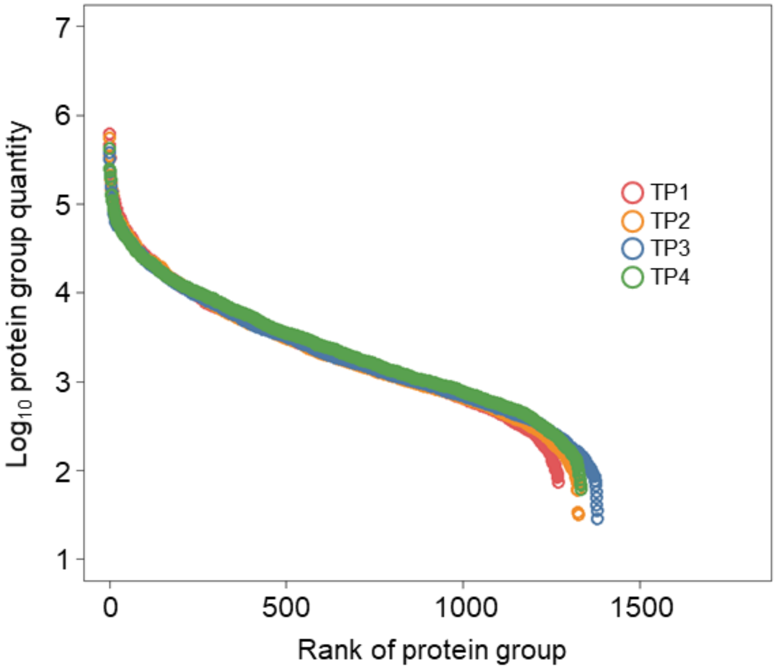

Supplementary Figure 2. Dynamic range. The plot depicts for each sampling time point the identified protein groups ranked by their abundance highlighting the dynamic range of proteins that can be quantified. Colors indicate the different sampling time points (TP1 – TP4).

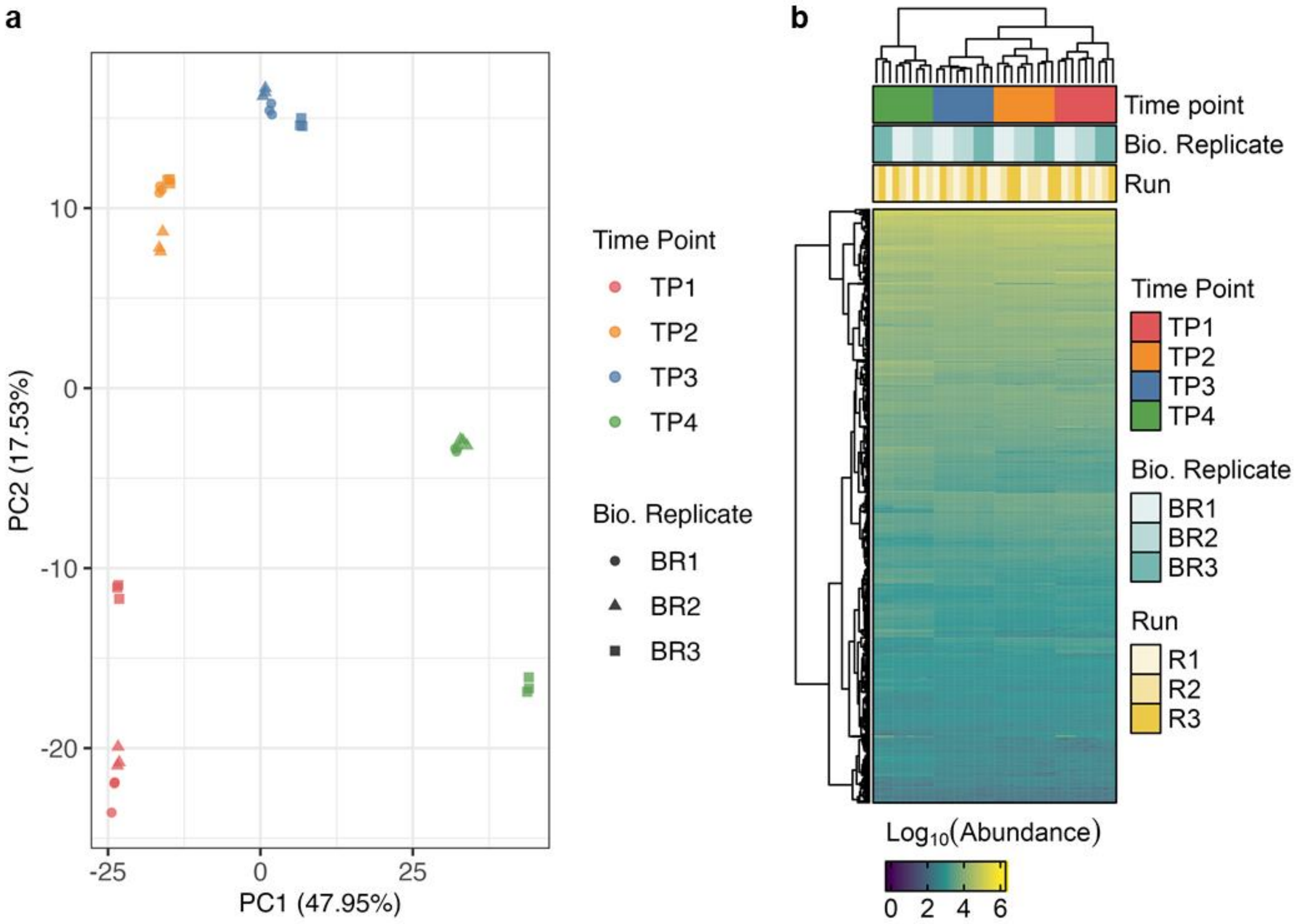

Supplementary Figure 3. Proteome profiles of *H. salinarum* NRC-1 measured with DIA/SWATH-MS. Four time points were sampled in a growth curve with TP1 - early exponential phase, TP2 - mid-exponential phase, TP3 - late exponential phase, and TP4 - stationary phase. Data were analyzed with the Spectronaut software. **(a)** Principal Component Analysis (PCA) of proteome profiles. Each marker represents a SWATH-MS run. Colors indicate the sampled time point and marker shapes the biological replicate. PCA plot was generated using the R stats::prcomp function and depicted with ggplot2. Biological and technical replicates cluster tightly while data sampled at different time points separate from each other; **(b)** Heat map of samples and log_{10} protein abundance clusters from the four sampled time points. Time points, biological replicates and SWATH-MS runs are colored for visualization purposes. Results were generated with ComplexHeatmap.

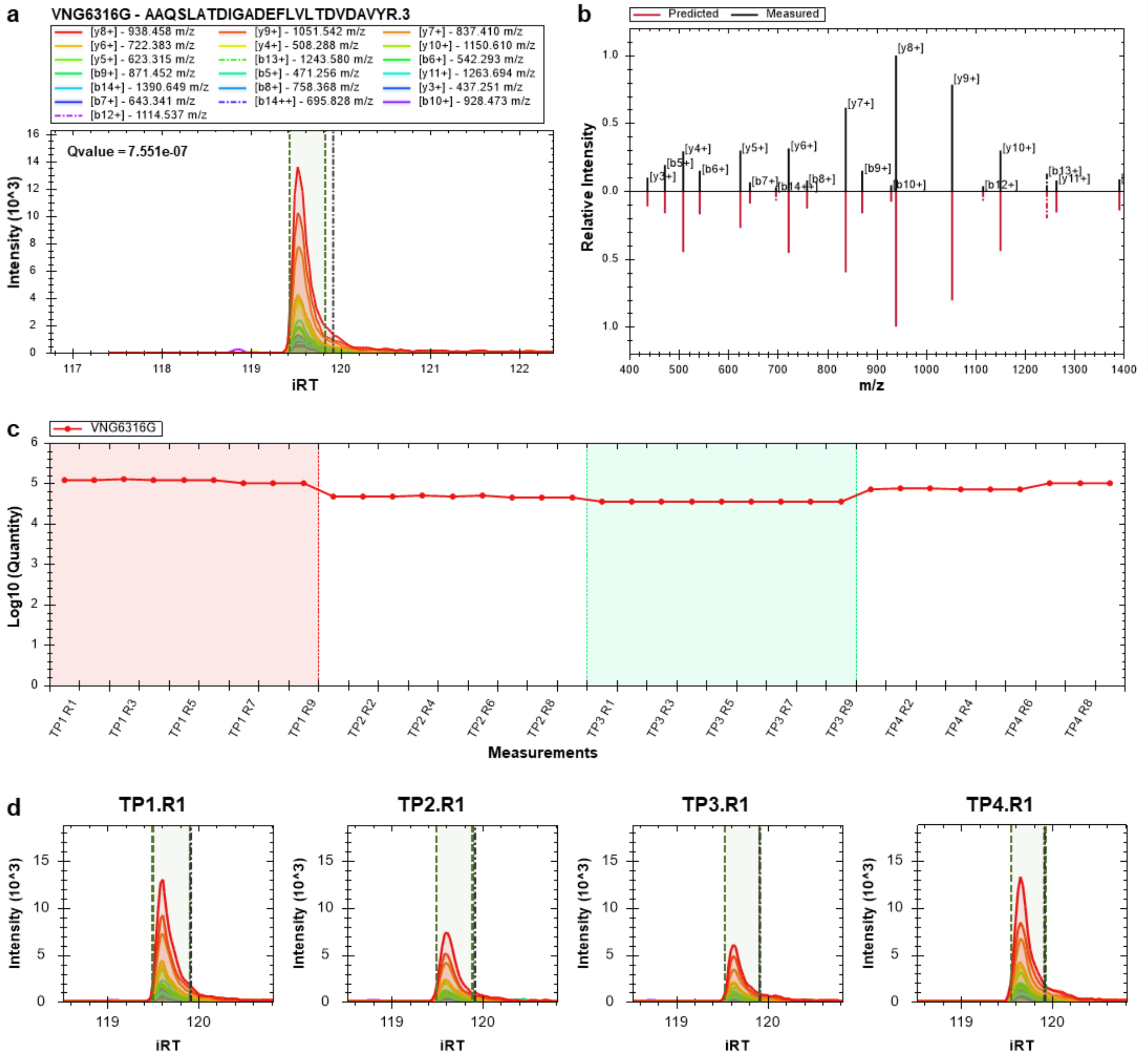

Supplementary Figure 4. VNG6316G shows transiently decreased protein abundance during exponential growth. (a) Extracted ion current chromatogram (XIC) of peptide AAQSLATDIGADEFLVLTDVDAVYR.3 from protein VNG6316G; **(b)** MS2 Intensity correlation plot of peptide AAQSLATDIGADEFLVLTDVDAVYR.3. The plot shows very good correlation of expected relative intensities in the library (red) and measured intensities of these fragment ions with DIA/SWATH (black). Well correlating fragment ions are depicted as solid lines, dotted lines indicate potential interferences; **(c)** Protein Profile of VNG6316G shows transiently decreased

abundance during exponential growth relative to the abundance in early exponential phase and stationary phase. Protein abundance in each MS run is depicted across four sampled time points (TP1 to TP4) with nine runs for each time point (R1 to R9, 3 biological and 3 technical replicates); **(d)** XIC plots of peptide AAQSLATDIGADEFLVLTDVDAVYR.3. across time points. Depicted is the first run (R1) from each time point highlighting the decreased abundance during exponential growth on the peptide level.

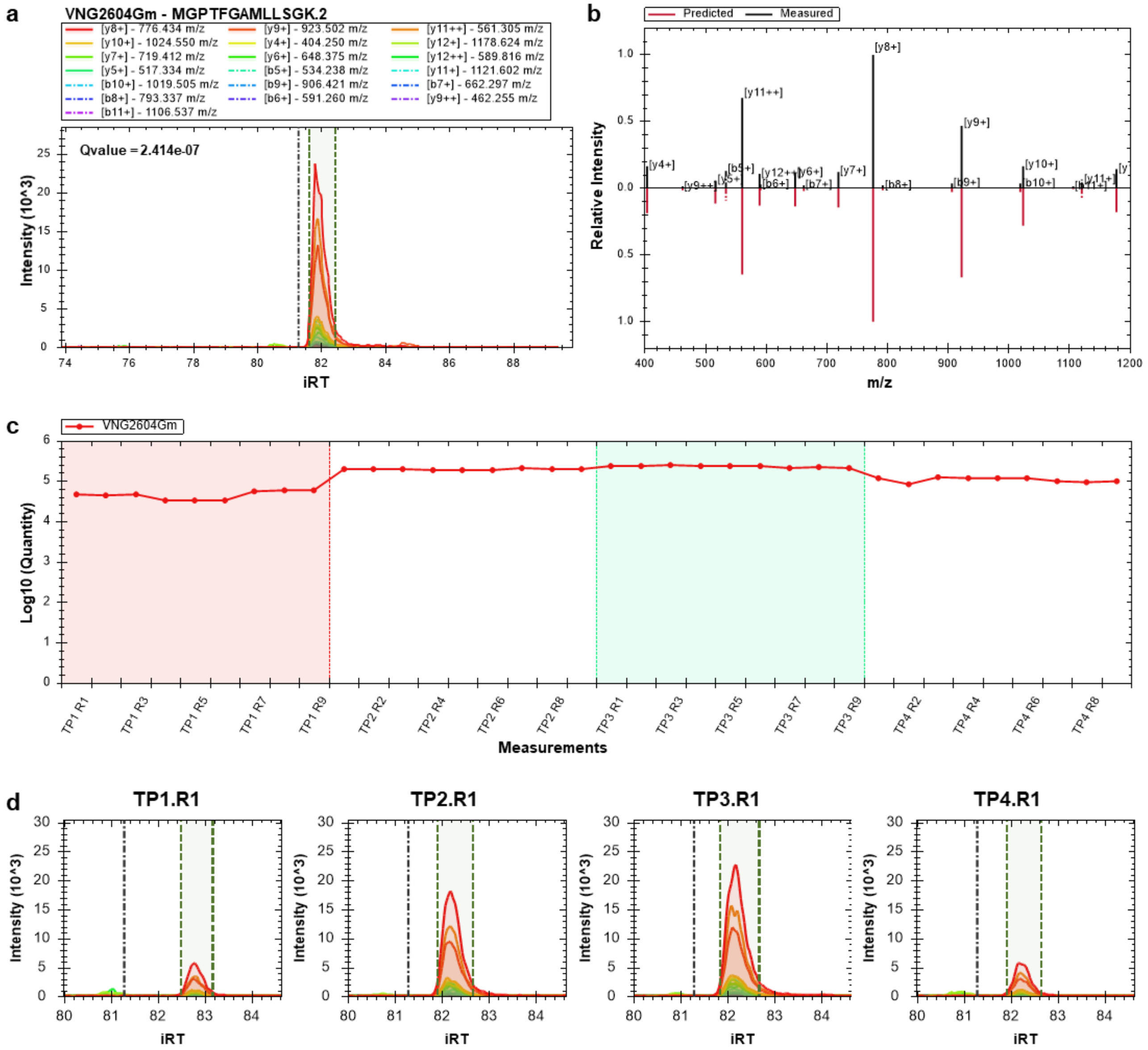

Supplementary Figure 5. VNG2604Gm shows transiently elevated protein abundance during exponential growth. (a) Extracted ion current chromatogram (XIC) of peptide MGPTFGAMLLSGK.2 from protein VNG2604Gm; **(b)** MS2 Intensity correlation plot of peptide MGPTFGAMLLSGK.2. The plot shows very good correlation of expected relative intensities in the library (red) and measured intensities of these fragment ions with DIA/SWATH (black). Well correlating fragment ions are depicted as solid lines, dotted lines indicate potential interferences; **(c)** Protein Profile of VNG2604Gm shows transiently elevated abundance during exponential growth relative to the abundance in early exponential phase and stationary phase. Protein

abundance in each MS run is depicted across four sampled time points (TP1 to TP4) with nine runs for each time point (R1 to R9, 3 biological and 3 technical replicates); **(d)** XIC plots of peptide MGPTFGAMLLSGK.2. across time points. Depicted is the first run (R1) from each time point highlighting the decreased abundance during exponential growth on the peptide level.

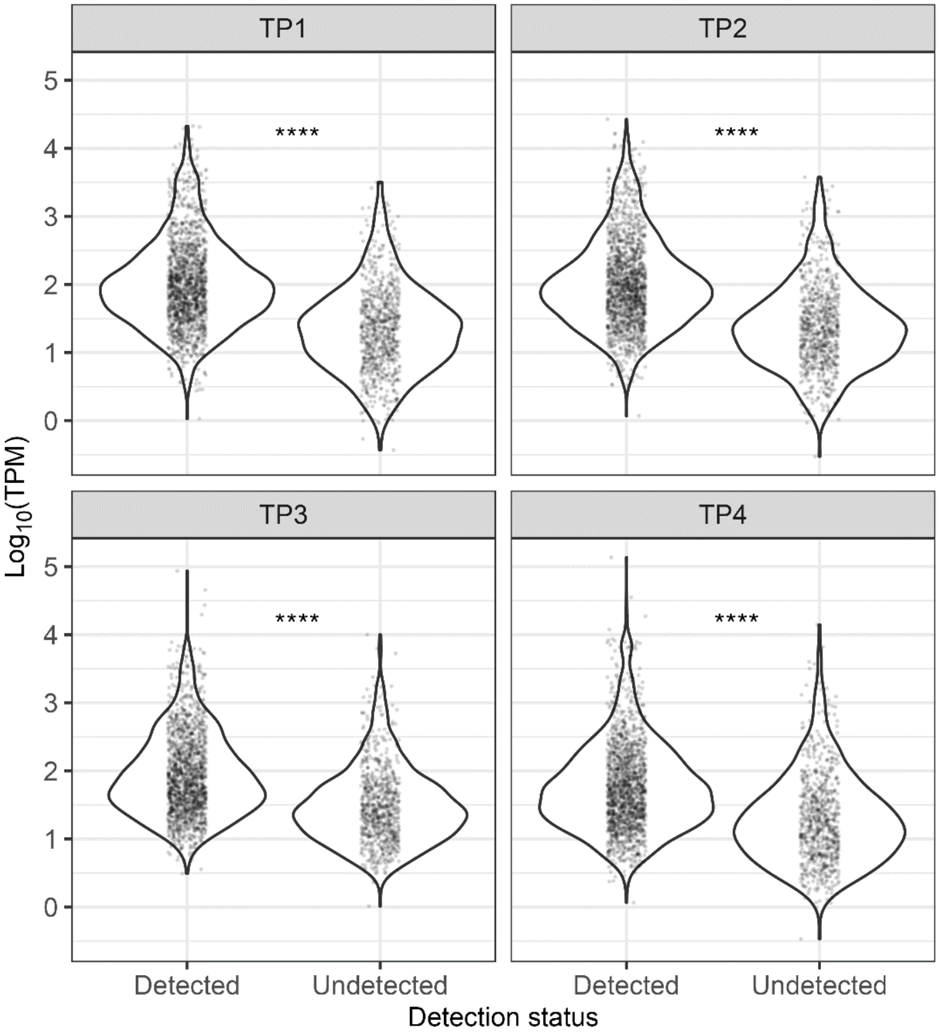

Supplementary Figure 6. mRNA abundance levels of detected and undetected proteins. Comparison of average mRNA abundance levels (log10-transformed transcripts per million) of detected and undetected proteins during batch culture growth using Student's t-test. **** denotes p-values < 0.0001. Each panel represents a sampled time point with TP1 for early exponential phase, TP2 for mid-exponential phase, TP3 for late exponential phase and TP4 for stationary phase.

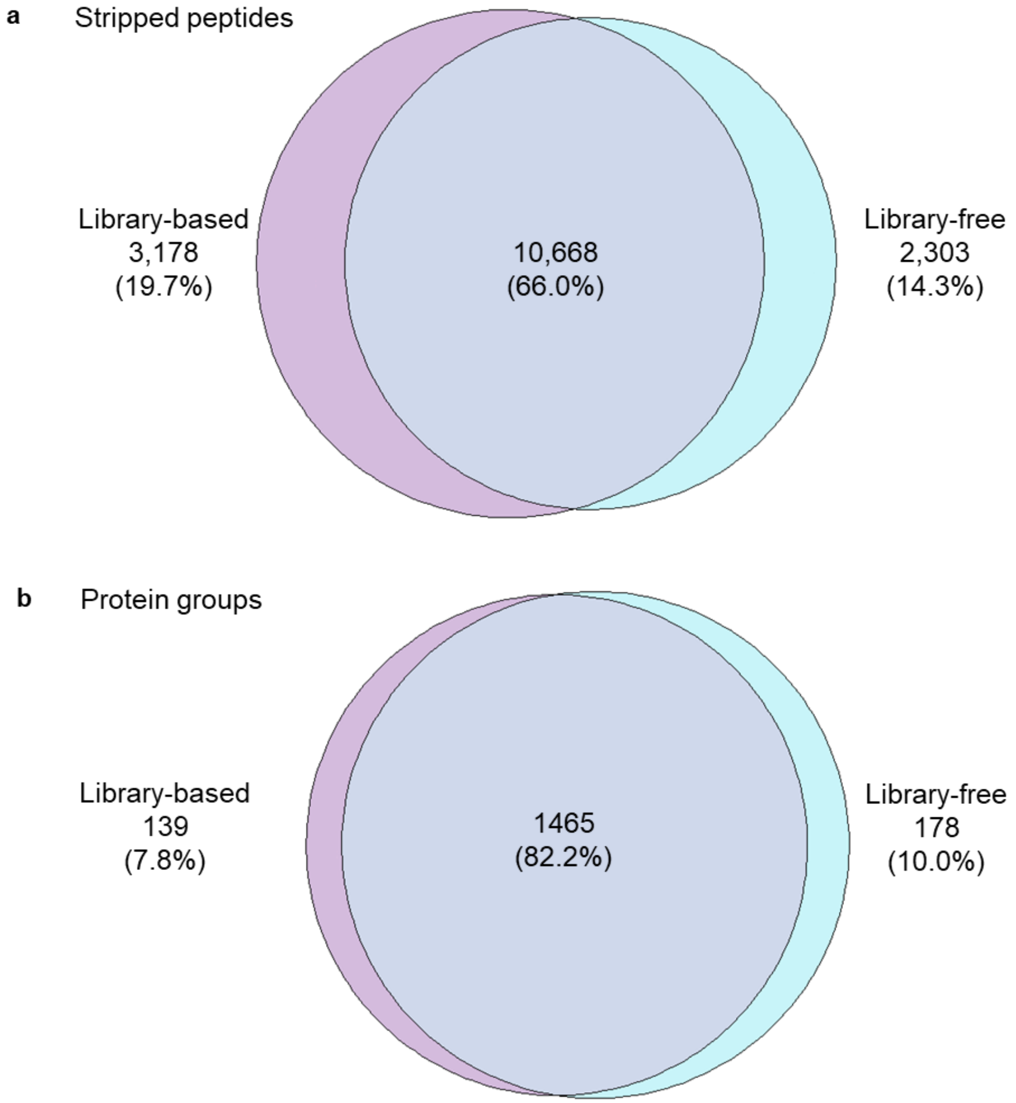

Supplementary Figure 7. Library-based and library-free analysis. The *H. salinarum* NRC-1 time-course data were analyzed with the developed spectral assay library in Spectronaut (library-based) and in addition with Spectronaut's directDIA option (library-free). Comparing the results on **(a)** the peptide and **(b)** the protein level showed more unique quantified peptides with the developed library in comparison to the library-free approach and comparable results on the protein level suggesting that a hybrid approach of library-based and library-free may further improve the number of peptide and protein identifications.
